# The impact of dual-tasking on mnestic performance in normal ageing

**DOI:** 10.1038/s41598-025-96784-z

**Published:** 2025-10-16

**Authors:** Giulio Contemori, Chiara Iorio, Mario Bonato

**Affiliations:** Department of General Psychology, University of Padua, Via Venezia 8, Padua, Italy

**Keywords:** Cognitive ageing, Human behaviour

## Abstract

Multitasking has become a necessity in our daily routines. Older people in developed countries are subject to ever-increasing cognitive demands, and the way they multitask has been raising significant interest. Recent research has focused on the clinical relevance of the motor-cognitive dual-task, partially neglecting the potential of concurrently using two cognitive tasks. Here, we devised a computer-based, cognitive-cognitive dual task to study the relationship between dual-task cost, age, and cognitive efficiency (MoCA) in a group of sixty-one healthy participants (aged 50–77 years). Participants were tested with a primary visual Memory task (free recall or forced choice) and a secondary phonemic Fluency task, either concurrently (dual-tasking) or non-concurrently (single task). As expected, primary and secondary task performance significantly decreased with concurrent task demands and increasing age. Age and cognitive load however did not interact: The dual-tasking cost in visual Memory and in phonemic Fluency was stable across the age range we investigated. Participants with higher mnestic costs had lower performance also in other measures of divided attention (e.g. TMT) while no correlation was found with the MoCA score. This might be compatible with the presence of a core ability allowing to split attention for the parallel processing of information with different nature. In conclusion, cognitive-cognitive dual-tasks provide a sensitive measure of non-pathological age-related changes in cognitive efficiency and could be used as baseline when developing more sensitive tests for the early detection of abnormal patterns possibly indexing cognitive impairments.

## Introduction

In recent decades, there has been a significant increase in media and technology usage, resulting in increasing demand for multitasking in daily life^1^. Older individuals, in particular, are encountering novel and more intense cognitive demands^2^. This phenomenon can be partially attributed to the widespread adoption of technology and to the substantial rise in retirement age, observed in virtually all developed countries since the mid-to-late 1990s^3^.

Multitasking performance varies significantly among individuals and is influenced by both situational and individual factors^4,5^. It is still debated whether multitasking relies on multiple specialized, domain-specific cognitive functions or on a singular, domain-general, cognitive resource^6–8^. The drops in performance characterizing multitasking are primarily attributed to the limitations of cognitive functions involved in performance monitoring, such as working memory, attentional and executive resources^6,7,9^.

Numerous studies have demonstrated that dual-task paradigms can effectively reveal deficits in cognitive functioning that are not evident under single-task conditions^10,11^. For example, the use of dual-task methodologies has been shown to be instrumental in identifying age-related differences in cognitive processing and multitasking abilities, highlighting the role of attentional and executive resources in managing concurrent tasks^12–14^. Furthermore, dual-task costs have been systematically linked to real-world (multitasking) performance, such as driving while conversing, underscoring the ecological validity of these structured tests^15,16^. An example of this is the Useful Field of View (UFOV) test, which involves identifying a central target while simultaneously detecting peripheral targets amid distractors. This test mimics the visual demands characterizing real-world scenarios, such as driving, where one must monitor the road ahead while being aware of peripheral hazards^17^. Performance on the UFOV test has been linked to handling traffic complexity and responding swiftly to road hazards. Reduced UFOV capacity is correlated with an increased risk of accidents, especially among older adults^18,19^.

Similarly, there is evidence that older adults who stop walking to reply to a question are at a greater risk of falls and injuries compared to those who can walk and talk simultaneously^20,21^. The performance decrements observed in controlled laboratory settings often reflect even greater challenges in real-world environments^22^. Thus, laboratory-based dual-task costs can also serve as a valuable proxy for assessing real-life multitasking abilities and, more broadly, evaluating individual cognitive efficiency in structured testing environments.

The way in which multitasking ability changes during ageing has been object of increasing interest. The Motor-Cognitive Dual-Task (MCDT) paradigm, which involves simultaneously performing a motor task and a purely cognitive task, typically testing working memory (WM), has been widely used to investigate age-related changes in multitasking ability^20^. MCDT has shown potential in distinguishing cognitive subtypes of dementia and other pathologies^23,24^. Despite its diagnostic potential, there has been limited research extending these findings to those clinical populations mostly affected by cognitive symptoms. This might explain why the Cognitive-Cognitive Dual Task (CCDT) approach is well-recognized as a valuable research tool but is comparatively less utilized in clinical practice, despite its sensitivity and ease of administration^25^. Computerized versions of the CCDT hold promise for uncovering subtle cognitive deficits that may not be detectable with traditional paper-and-pencil tests. Their potential has been fully exploited in domains such as post-stroke asymmetries in spatial processing^26,27^ but has been also used for investigating the characteristics of healthy aging^28–30^.

One significant challenge clinicians face when designing demanding tasks is the variable performance observed among healthy participants. Dual-tasking (DT) methodologies allow us to modulate the difficulty of tasks and measure the “cost”—the difference between performance in basic and complex cognitive contexts. This DT setup mimics real-life cognitive scenarios and limits the use of compensatory strategies. Research shows that older adults exhibit performance deficits when diverting attention from irrelevant stimuli^31,32^, switching between cognitive tasks^33^, and resuming a primary task after an interruption^34^. Additionally, multitasking abilities are linked to biological and cognitive markers associated with pathological aging^24,35^.

A simple test like the Mini-Mental State Examination (MMSE)^36^ has been proven sensitive enough for detecting performance drops a decade before a clinical diagnosis of neurodegenerative disease^37^. Yet we know that MMSE is not particularly sensitive^38^, particularly when it is administered as a stand‐alone single‐administration test for a quick assessment^39,40^.

Here, we present proof-of-concept data from a dual-task manipulation that requires verbal responses during memory encoding. We measured baseline and dual tasking performance in a group of healthy adults across various ages to investigate the relationship between CCDT costs, cognitive efficiency, and aging in a non-clinical context, resulting in the definition of the so-called individual aging cognitive trajectories. Our approach integrates a computer-based visual memory task with a phonemic fluency task. This dual-task paradigm involves the concurrent implementation of a phonemic fluency task during the encoding of visual stimuli. Participants were required to either recall (free recall test) or recognize (forced choice recognition test) previously shown images. The phonemic fluency task was performed during the encoding of visual stimuli and then each image had to be either named (free recall test) or selected among four possible similar alternatives (forced choice recognition test).

In a parallel project^28,30^ we implemented the same idea by using an online procedure. While the primary task was almost identical to the present one, the concurrent task differed, as it required the monitoring of an auditory stream of letters. That methodological choice was determined by the fact that the task was self-administered online while here the presence of an experimenter made it possible to implement phonemic fluency as concurrent task, which is characterized by the possibility to continuously monitor the attentional engagement by the participants and precisely quantify their performance.

According to the “dual-process signal detection model” (DPSD), the ability to recognize previously encountered objects depends on two different processes, namely recollection and familiarity^41^. Recollection is characterized by the retrieval of specific contextual details associated with a previously encountered event or item. This process is often described as a qualitative, threshold-based mechanism where the retrieval includes rich, episodic details such as the time, place, or associated thoughts and emotions related to the initial encoding of the memory. Recollection allows individuals to mentally re-experience past events with a high degree of detail and confidence. Familiarity, on the other hand, provides a general sense of prior exposure to a stimulus without retrieving specific contextual details. It is considered a more quantitative, continuous process compared to recollection, as it reflects the strength of the memory signal, “the feeling of knowing”, even when the precise context cannot be recalled^42^. Evidence suggest that decreased mnestic performance characterizing healthy ageing mostly (or only) concerns recollection, while familiarity remains intact^43^. In contrast, cognitive decline and dementia impair both recollection and familiarity^44,45^. In this respect, a task tapping mostly on recollection might be a good option when, as in this case, the testing does not involve pathological participants and is focused on determining cognitive trajectories for healthy ageing. Since free recall is based more on recollection and forced choice with similar foils on familiarity^46,47^ we expect, with increasing age, performance decreasing more in free recall than in forced choice recognition. We also hypothesised that this age by response modality (free recall vs. forced choice) interaction would increase in amplitude as the secondary task causes a shallow encoding of visual information^48^. Previous research showed that age-related effects on memory are mediated by retrieval and by encoding processes associated with frontal control and working memory^49^. We expected decreased performance due to aging and dual tasking requirements. We were also particularly interested in testing whether the dual-task (DT) cost on visual memory and fluency would decrease or remain stable with increasing age, as we found in the previously described online variant with a different concurrent task^28,30^. Finally, we were also interested in measuring performance under divided attention across different cognitive domains. This was measured using the Trail Making Test (TMT)^50,51^ and Interference Memory Tests^52^. Finally, we also expected that participants with higher memory DT cost had a worse global cognitive status as measured by the Montreal cognitive assessment (MOCA)^53–55^.

## Methods

### Participants

61 Italian-speaking volunteers aged between 50 and 77 years (32 women), were recruited from associations, parishes, clubs, and recreational groups for elderly people in the Carpi area (Modena, Italy). The sample was selected to have an even distribution in terms of age (approximately 20 participants for each decade). The inclusion criteria were normal or corrected for normal vision, normal hearing, and absence of neurological pathologies, psychiatric disorders, and a normal score on MoCA^55^. The persons who took part in the study were White, with a mean age was 65 years (range 50–77) and a mean education of 11 years (range 5–17). All participants gave their informed written consent prior to inclusion in the experiment and received no compensation for their participation.

### Procedure

The experimental protocol consisted of an initial anamnesis, a battery of neuropsychological tests, and a computer-based experimental task. Participants were seated at a table in a sufficiently illuminated, quiet, and comfortable room. The computer showing the stimuli for the single and dual-task was placed in front of them, at about 60 cm. Reaction times were not recorded and the experimenter (CI) reported the answers in a response sheet. Therefore, participants were not required to know how to use the computer. The order of the tests was kept constant for all the participants. The letters in the phonemic fluency task and the categories of stimuli for the memory test were counterbalanced between subjects. Participants were specified from the beginning that they could interrupt the experimental procedure at any time and withdraw their consent to the use of their data. The procedures were approved by the Ethics Committee for Psychological Research of the University of Padua (prot. 2491), and performed in accordance with the Declaration of Helsinki.

### Cognitive assessment

First, we administered the Italian version of the MoCA^55^ as an initial screening test. Following this, we administered four subtests from the “Esame Neuropsicologico Breve” (ENB-2) battery, a standardized neuropsychological assessment, following the instructions for the Italian version^52^. These subtests included: the phonemic fluency test (utilizing the letters C, P, and S according to the protocol), the Trail Making Test A and B (TMT-A/B), the Interference Memory test with 10 and 30 s intervals, and the immediate and delayed Story Recall Test (SRT). The interval between the immediate and delayed recall for the SRT was 4 min^52^. Each participant was individually examined in a single experimental session lasting approximately 45–60 min (Table 1).

**Table 1:** Summary of the experimental procedure/task order.

(1) MoCA^55^
(2) Phonemic Fluency (2 letters)
(3) Computer-based visual Memory task (single task): first set of images
Explanation of the task + example trial
Presentation of the images (10) to be memorized
Free recall
Four-alternative forced choice
(4) Computer-based visual Memory task (dual-task): second set of images
Explanation of the task + example trial
Presentation of the images (10) to be memorized + concurrent phonemic Fluency
Free recall
Four-alternative forced choice
(5) Interference memory 10 and 30 s test
(6) Story recall test (SRT): immediate recall
(7) Computer-based visual Memory task (single task): third set of images
Presentation of the images (10) to be memorized
Free recall
Four-alternative forced choice
(8) Story recall test (SRT): delayed recall
(9) TMT-A/B

### Computer-based visual Memory task (CCDT)

**Fig. 1:**
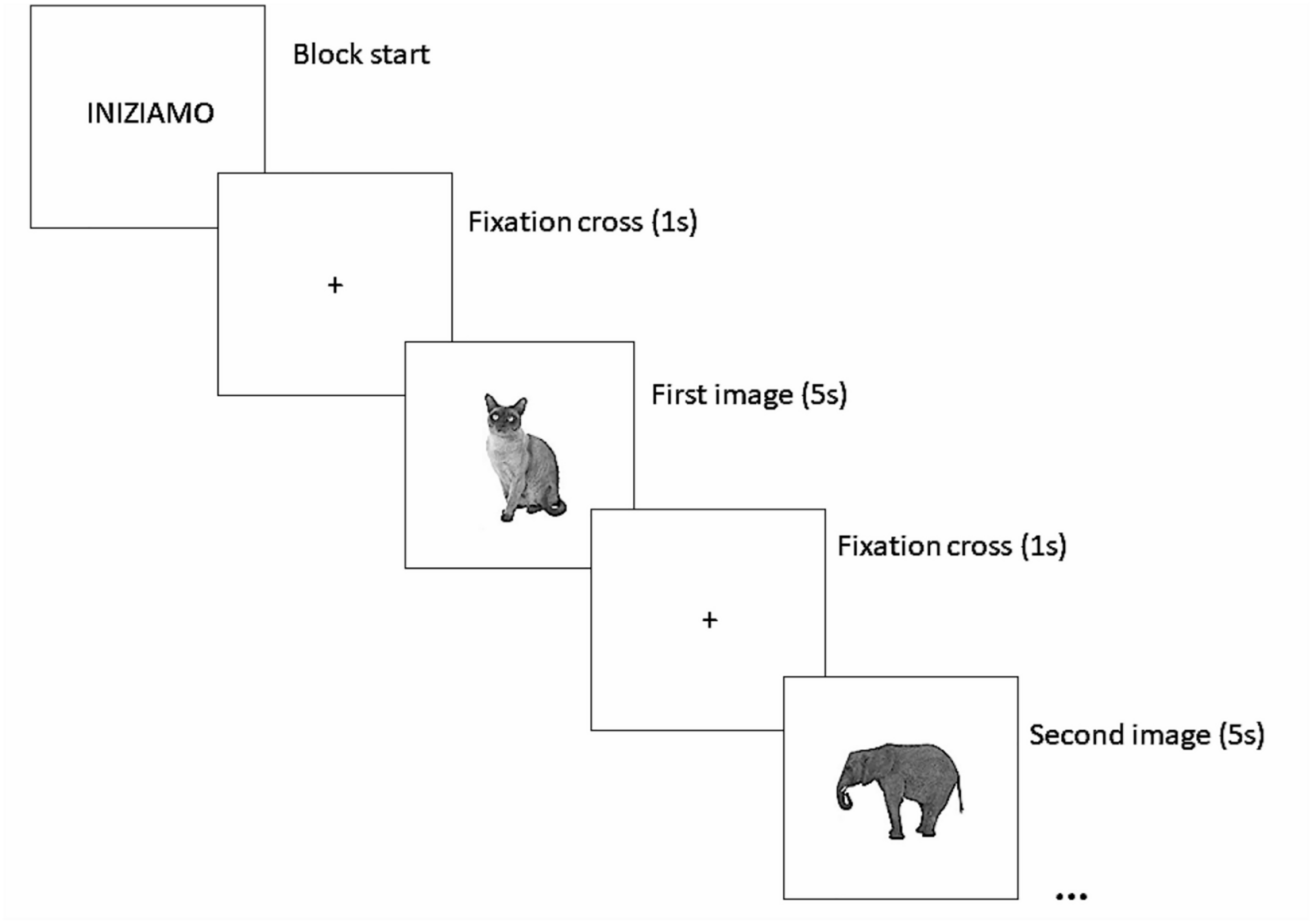
Computer-based visual Memory task. A representative example of the memorisation phase (animals). “INIZIAMO” means "Let’s start"

Participants performed the visual Memory task three times during the session: twice in the single-task condition and once in the dual-task condition. In the single task condition (baseline), participants were asked to memorise 10 images, sequentially presented on a computer screen (Fig. 1).

Participants were informed that they would first be asked to name the elements they remembered (free recall) and later to recognise the correct image among alternatives. During the dual-task block (DT), participants had to perform a phonemic Fluency task simultaneously to the visual presentation (identical to the single task). A second block of single task (ST2) was presented during the 4’ interval between the immediate and deferred recalling of the Story recall test^52^. Images (black and white) of the series to be memorized were selected from a database standardized for the Italian population^56^. There were three series, each encompassing ten elements belonging to the same semantic category (animals, vegetables, and kitchen utensils). Each image was presented in the centre of the screen for five seconds, interspersed with a central fixation cross that lasted for one second. Phonemic Fluency was measured during the entire block duration (one minute). In this way the time available for phonemic Fluency was identical to the time allowed during the baseline condition. The order of the slides within the series was the same for all participants. In the free recall participants had to verbally report the items they remembered. 

In the forced recognition phase, which followed the free recall, each slide contained four different images belonging to the same category of the target item. For example, if the item to be remembered was a horse, the images of four horses were shown. The position of the target stimulus among the four alternatives was randomized between trials. The arrangement of the images within the slide and the order in which the sequence was presented (different from the initial series) were fixed and identical for all participants. In the recognition phase, there were no time limits and participants were asked to select the most plausible stimulus among the four alternatives provided. The baseline session and the dual-task phase were preceded by three example trials. As ST performance was measured both before and after the DT block (Table 1, points 3 & 6) average performance was used for the analyses after having ensured that the two conditions did not differ (see “Results” section). This approach prevented the more challenging DT block from being conducted first and minimized potential practice effects between the ST and DT conditions, as the ST was performed both at the beginning and end, with accuracy scores averaged accordingly.

### Phonemic fluency task

The phonemic Fluency task has been administered and corrected according to the procedures described in the original publication^52^. Each participant performed the task for two different letters for 60 s each for the single task condition and for a third letter in the dual-task condition during the memorisation of the images. The order of the three target letters was randomly assigned among the participants. Phonemic Fluency was chosen as a secondary task as it is based on recollection ability and therefore may cause greater interference with the primary task than other tasks involving different mechanisms. Furthermore, the verbal response modality prevents verbalisation and verbal rehearsal of visual stimuli.

### Data analysis

The analyses were performed with the R software package^57^. Specifically, we fitted a generalized linear mixed model for the binomial memory data using the "glmer()" function from the “lme4” package^58^. To ensure model convergence and avoid multicollinearity issues, we centered the age variable. Both the main effects and interactions were later examined with a Type III Wald chi-square test using the "Anova()" function from the “car” package^59^. In our GLMM, we included Response Type (free recall vs forced choice), Cognitive Load (single vs dual-task), and Age of participants as between-subject variables. Participants were added as a random factor in the model. To evaluate effect sizes, we employed the partR2 package^60^, which computes semi-partial R^2^ values specifically for mixed models. These values provide an approximation of the variance explained by individual predictors, offering a practical alternative to traditional effect size measures that are not directly applicable in the context of mixed modelling.

A similar analytical approach was applied to the phonemic Fluency. In this case the generalized linear mixed model based was based on a Poisson distribution which is suitable for analysing count data. The model included the type of Cognitive Load (single vs dual-task), and Age of participants as between-subject variables, and the participants as a random factor. The omnibus test was a type III Wald chi-square test.

Moreover, we calculated Bonferroni corrected post-hoc against zero for the two response modalities with the “glht” function of “multicomp” package^61^. In the final section of the results, we also reported several Pearson’s correlation coefficients calculated with the "cor.test" function. We examined correlations between the visual Memory dual-task cost for the two response modalities with cognitive test scores (Trail Making Test [TMT], Montreal Cognitive Assessment [MoCA], Interference Memory at 10 and 30 s), and phonemic Fluency cost. Phonemic Fluency dual-task cost was also correlated with the cognitive test scores.

The dual-task cost for both the images and the phonemic Fluency was calculated as the difference between the number of items/words retrieved in single-task minus performance in dual-task conditions. Since the single-task condition was administered twice, dual-task cost was computed relative to both baseline blocks, and these two values were subsequently averaged. Positive values indicated a cost (for the visual Memory task: more errors in the dual-task conditions; for Fluency: less words), whereas negative values indicated a gain. To identify common patterns in strategies employed during dual-tasking, we also calculated correlations between the dual-task cost in phonemic Fluency and the dual-task cost in memory tasks (separately for free recall and forced choice).

The probability of obtaining at least one statistically significant correlation out of 17 by chance was 0.6, which we consider acceptable in the context of this study^62^.

## Results

We report the results in the following order: visual Memory task, phonemic Fluency, correlation between the cognitive test batteries and dual-task cost.

### Computer-based visual memory task

For the computer-based visual Memory task, there was no significant difference between the single task block performed at the beginning and at the end of the testing procedure (mean difference for forced choice = − 0.13, *t*(*60*) = − 0.905, *p* = 0.37; mean difference for the free recall = − 0.38,* t*(*60*) = − 1.926, *p* = 0.059). Data from the two blocks were therefore aggregated in the subsequent analysis. Table 2 provides the means and standard deviations for each condition in the visual Memory task.

**Table 2:** Descriptive statistics for image recognition (memory score) in the visual Memory task.

Response type	Condition	Visual memory task: Memory score (range 0–10)	sd
Forced	Dual	7.656	1.940
Free	Dual	5.148	2.048
Forced	Single	9.230	0.699
Free	Single	7.566	1.054

**Fig. 2:**
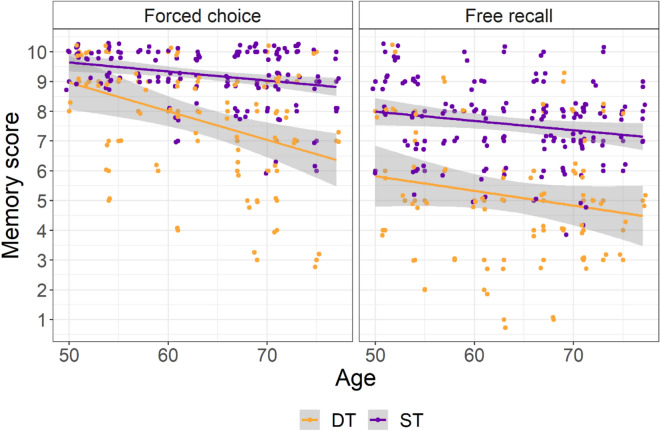
Visual Memory task. The number of correctly reported items (memory score) is reported as a function age (in years) separately for Response Type (forced choice on the left vs. free recall on the right) and Cognitive Load (DT Dual-Task vs ST Single-Task). For visualization purposes a 0.3 horizontal and vertical jitter has been added. For the single task, data from both repetitions are reported.

We performed a Type III Wald chi-square test on the binomial outcome for each item in the visual Memory task (memory score). There was a significant main effect of Response Type (W(1) = 202.129, *p* < 0.001, partR^2^ = 0.065), with higher scores observed for the forced choice (see Fig. 2). Additionally, there was a significant main effect of Cognitive Load (W(1) = 171.660, *p* < 0.001, partR^2^ = 0.045), with the single task resulting in higher scores compared to the dual-task, confirming the expected negative impact of a CCDT during encoding. Age also resulted in a significant effect, with the average number of correctly remembered items decreasing with increasing age of the participants (W(1) = 16.035, *p* < 0.001, partR^2^ = 0.01). Furthermore, the interaction between Response Type and Age was significant (W(1) = 8.812, *p* = 0.003, partR^2^ < 0.001). Crucially, the Cognitive Load X Age interaction was not significant (W(1) = 0.330, *p* = 0.566, partR^2^ < 0.001), indicating that the impact of dual-tasking on mnestic performance did not differ across the age range. The Response Type X Cognitive Load interaction was also not significant (W(1) = 1.165, *p* = 0.280, partR^2^ = 0.007) as well as three-way interaction Response Type X Cognitive Load X Age (W(1) = 0. 0.181, *p* = 0.671), partR^2^ < 0.001, indexing a similar impact of age on performance under load for both tasks. A ceiling effect for younger participants in the easiest condition was visible (Fig. 2, left panel, upper left corner).

### Phonemic fluency

**Fig. 3:**
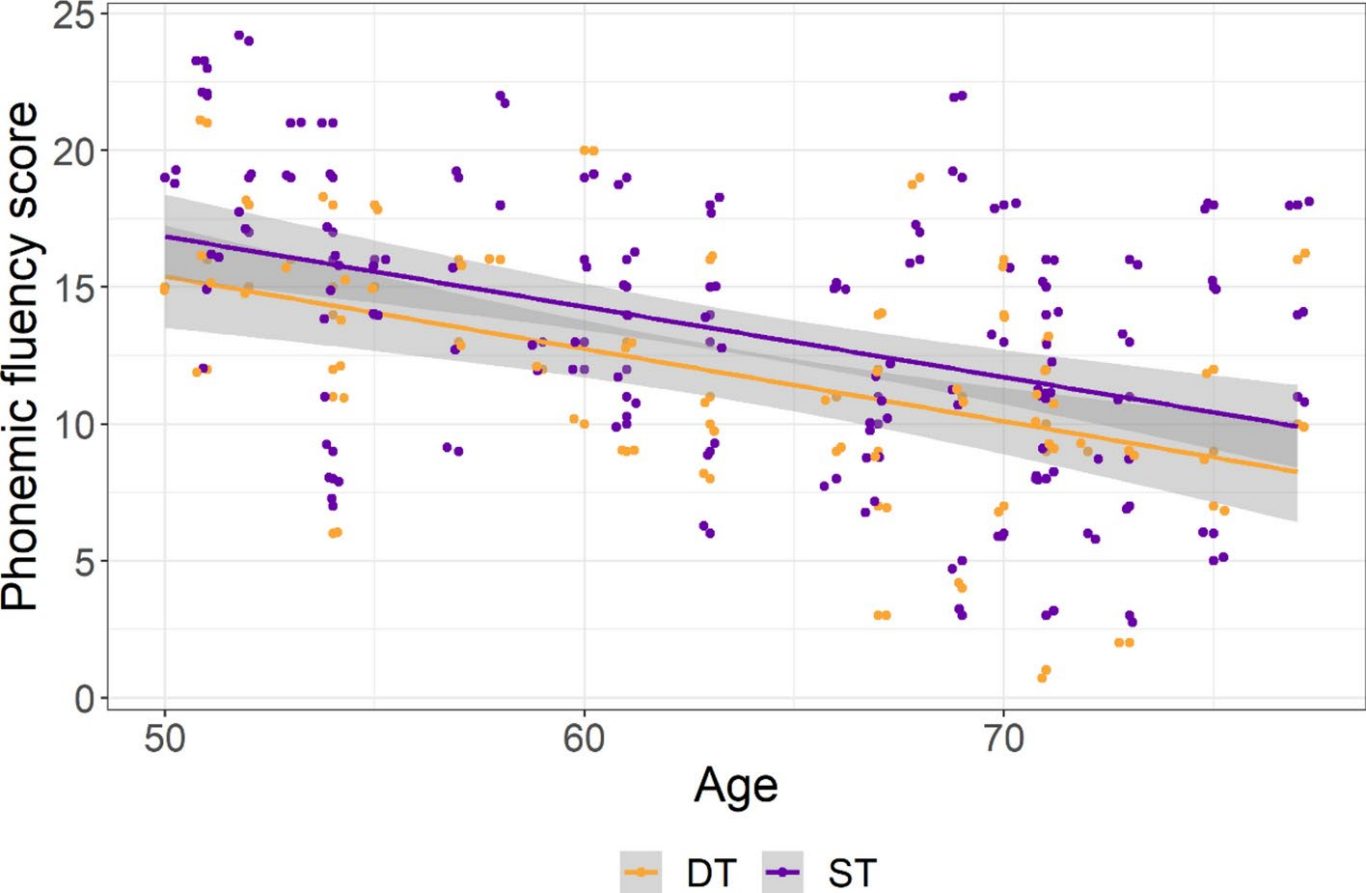
The phonemic Fluency performance reported as a function of age (in years) shown separately for the two levels of Cognitive Load (dual-task DT vs single task ST). For visualization purposes a 0.3 horizontal and vertical jitter has been added. For the single task, data from both repetitions are reported.

For phonemic Fluency, there was no significant difference between the single task block performed at the beginning and at the end of the testing (mean difference = 0.705, *t*(*60*) = 1.734, *p* = 0.09), and therefore data were aggregated for the subsequent analysis. Type III Wald chi-square test for the phonemic Fluency score showed a significant effect of Cognitive Load (W(1) = 8.092, *p* = 0.004, partR^2^ = 0.046) with more words (13.4) being produced during the single task (e.g. without having to memorize images) than during the dual-task (11.8). Age had a significant negative effect, indexing that the older the participant, the fewer words were produced (W(1) = 18.578, *p* < 0.001, partR^2^ = 0.009). Similar to the visual Memory task, the Cognitive Load X Age interaction was not significant (W(1) = 0.306, *p* = 0.58, partR^2^ = 0.003), (See Fig. 3).

### Correlations between cognitive tests and memory dual-task costs

**Fig. 4:**
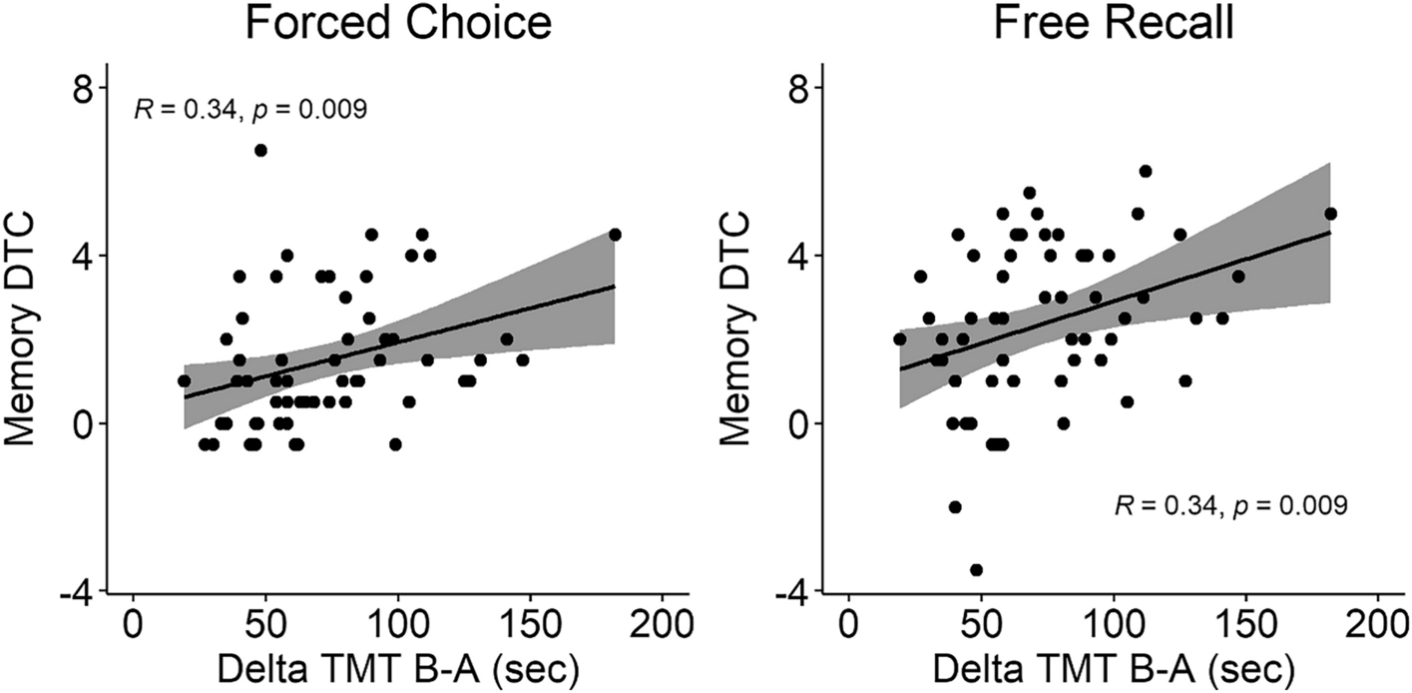
Significant positive correlations between the ΔTMT (in seconds) and the computer-based visual Memory dual-task cost DTC (positive values = more mnestic errors under dual-tasking) are reported separately for the forced choice (left panel) and the free recall (right panel) Response Type.

The correlations were calculated using a single score for each individual, costs for the visual Memory task, global score for MoCA and RTs for TMT. Costs were calculated by averaging together the visual Memory cost based on the first and second single task baseline for each individual. A similar approach was taken for the phonemic Fluency. The correlation between the cost in the forced choice and the cost in the free recall was not significant (*r*(59) = 0.049, *p* = 0.706). Next, we analysed the correlations between the cognitive tests and the memory costs. For the TMT differential scores ΔTMT (completion time TMT-B minus TMT-A, in seconds) were computed for all participants except two who had incomplete data. ΔTMT was positively correlated with the memory dual-task cost for free recall (*r*(57) = 0.34, *p* = 0.009) and forced choice (*r*(57) = 0.34, *p* = 0.009) (Fig. 4) indicating that the participants who made more errors in the Memory task were particularly slow in TMT B compared to TMT A.

**Fig. 5:**
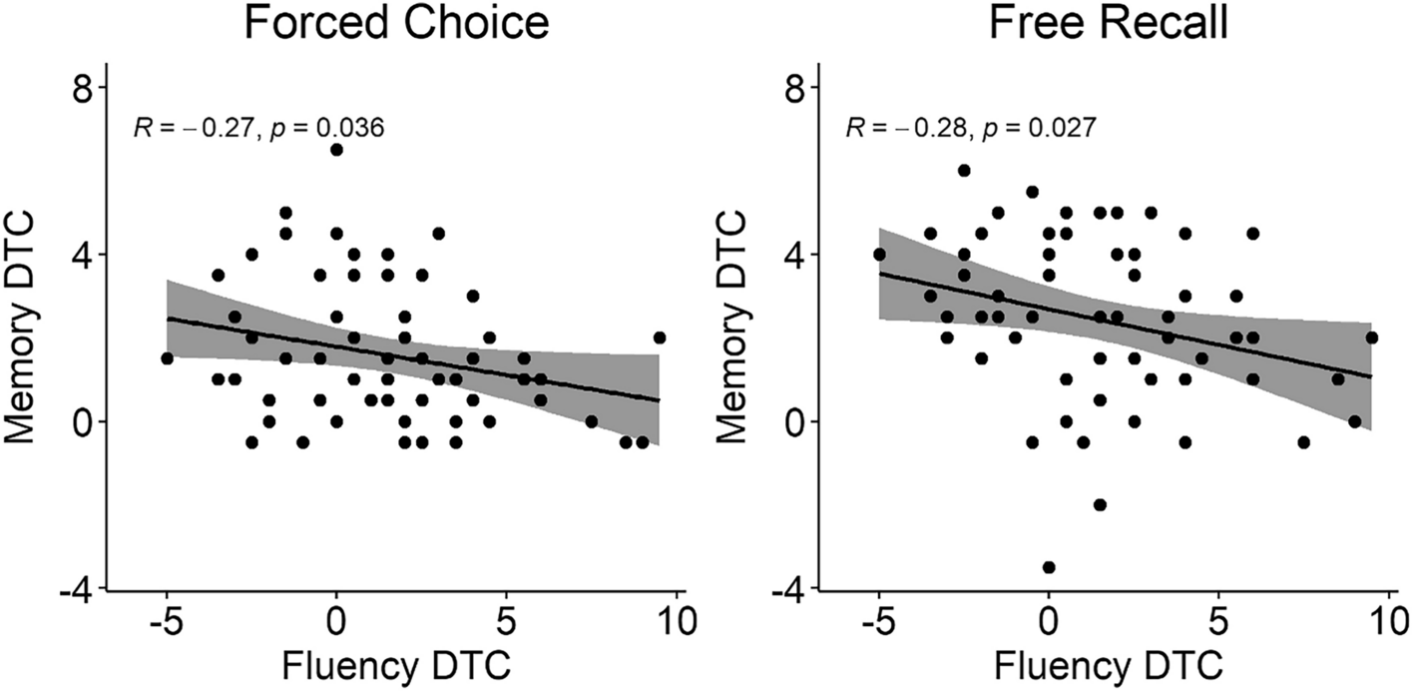
Significant negative correlations between the phonemic Fluency dual-task cost (Fluency DTC) and the visual Memory dual-task cost (Memory DTC) are shown separately for the forced choice (left panel) and the free recall (right panel) Response Type.

The Pearson correlation between phonemic Fluency cost and visual Memory costs was negative and significant for both the forced choice (r(59) = − 0.269, *p* = 0.036), and the free recall (r(59) = − 0.283, *p* = 0.027) (Fig. 5), suggesting a resource sharing/performance trade-off between the two concurrently performed tasks (higher cost in the visual Memory associated with lower cost in the Fluency and viceversa).

**Fig. 6:**
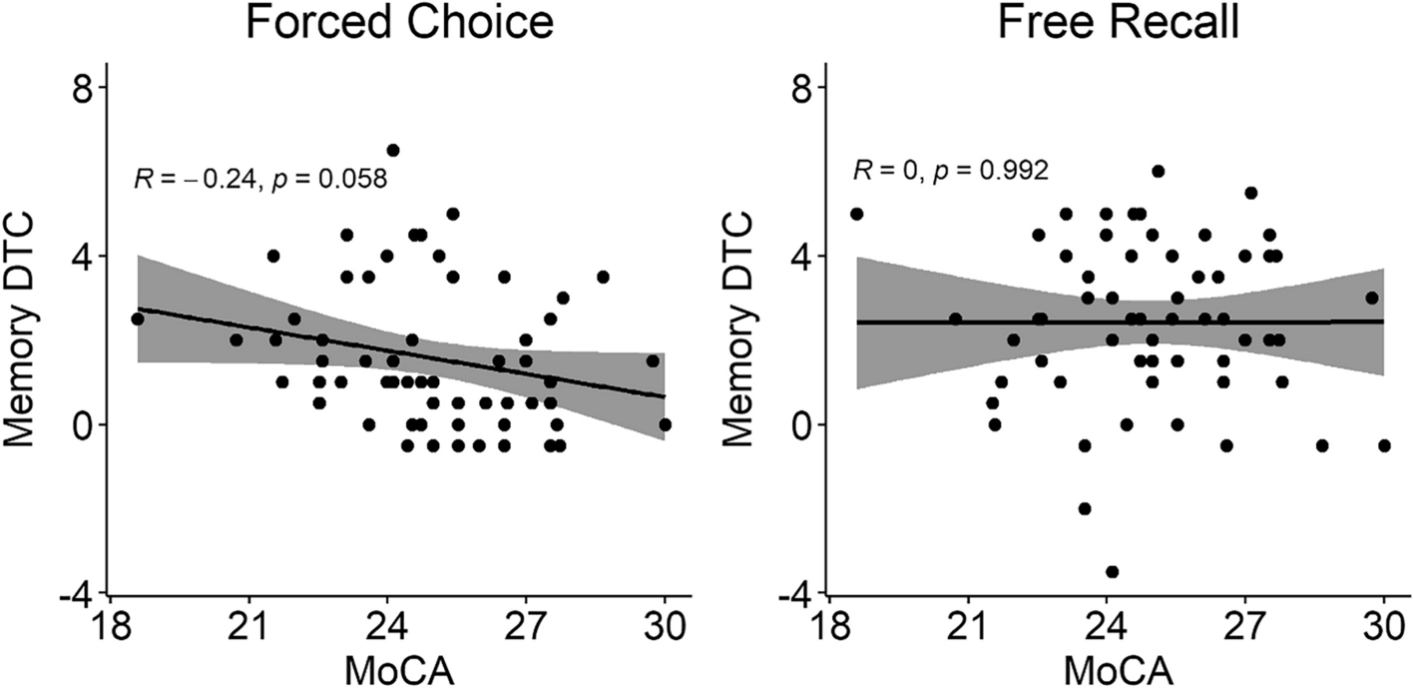
Nonsignificant correlations between the MoCA score and the visual Memory dual-task cost are shown separately for the forced choice (left panel) and the free recall (right panel) Response Type.

In contrast, the correlation between MoCA score and the visual Memory dual-task cost was not significant for the free recall (r(59) = 0.001, *p* = 0.992) nor for the forced choice Response Type (r(59) = − 0.245, *p* = 0.058) (Fig. 6).

The phonemic Fluency was not correlated with ΔTMT completion time (*r*(57) = − 0.19, *p* = 0.14) nor with MoCA (r(59) = 0.14, *p* = 0.28). On the contrary, the Interference memory significantly correlated with the cost in the visual Memory forced choice condition for both the 10 s (*r*(59) = − 0.366, *p* = 0.004), and the 30 s (*r*(59) = − 0.315, *p* = 0.014) task versions. For the free recall neither version was significant: 10 s (*r*(59) = 0.029, *p* = 0.825), and 30 s (*r*(59) = 0.214, *p* = 0.097).

Performance in the Story Recall Test significantly correlated with the cost in the visual Memory forced choice condition for the immediate (*r*(59) = − 0.314, *p* = 0.014), but not for the delayed (*r*(59) = − 0.234, *p* = 0.069) version. For the free recall neither version was significant: immediate (*r*(59) = − 0.07, *p* = 0.593), and delayed (*r*(59) = 0.002, *p* = 0.986).

In short, the detrimental impact of dual-tasking in the visual Memory task was associated with higher cost/lower performance also in other tasks, both mnestic (memory with interference, Story Recall) and non-mnestic (TMT).  In contrast, no correlation emerged with the outcome of the MoCA cognitive screening. Phonemic Fluency correlated negatively with the memory cost, indexing a trade-off in allocating resources for task execution.

## Discussion

By using a visual Memory dual-task which newly combines a forced-choice/free recall visual memory task with a phonemic Fluency task, we explored the relationship between accuracy dual-task cost, cognitive efficiency, and ageing in a nonpathological population. Then we correlated the memory dual-task cost for the two response modalities with cognitive test scores (Trail Making Test [TMT], Montreal Cognitive Assessment [MoCA], Interference Memory at 10 and 30 s), and with phonemic Fluency cost. Phonemic Fluency dual-task cost was also correlated with the cognitive test scores.

Mounting evidence suggests that the cost of the motor-cognitive dual tasks is predictive of dementia progression^63^ and could also be useful for differential diagnosis^24^. In contrast, most potential applications of the CCDT have not yet been investigated. For example, little is known about the interplay between cost in CCDT and cognitive abilities in healthy old adults.

In the present study, Dual-task requirements negatively impacted both phonemic Fluency and the number of visual items correctly recognized. Also, the negative impact of increasing age upon performance has emerged very clearly in both tasks, together with a performance trade-off suggesting that the same resources were allocated preferentially to one or another task. This suggests that, despite their difference, phonemic Fluency taps on resources overlapping with those involved in the encoding of visual images.

We selected a verbal task rather than a drawing task because it could be performed concurrently with image viewing. Additionally, using a concurrent verbal response modality minimized the likelihood of participants engaging in verbalization or rehearsal of visual stimuli during the task, thereby forcing participants to focus on visual encoding.

Choosing a phonemic Fluency task over a semantic Fluency task further reduced potential interference with the encoding process, particularly when considering that the images were presented in categorical groups. While a performance cost was expected in the dual-task paradigm, it is important to note that the tasks involved distinct cognitive domains. This separation likely helped to mitigate the extent of cross-task interference. For example, incorporating a semantic Fluency or design task might have imposed a higher cognitive load, potentially resulting in a greater dual-task cost with increasing age. However, it remains unclear to what extent increasing task difficulty is the most suitable approach to enhance sensitivity in detecting Alzheimer’s disease (AD).

 The manipulation used in this study revealed a relatively stable dual-task cost across ages. Future research could benefit from combining this approach with other methods that, by contrast, result in a pronounced impact of age on cognitive cost. Notably, the often described increase in multitasking costs with age did not emerge, replicating the findings from a parallel project^28,30^. These findings deviates from the general consensus in the literature, which often reports prominent age-related decline in multitasking abilities, although this is more evident when response speed, rather than performance accuracy, is used as the dependent variable (see Verhaeghen and Cerella^13^ for a comprehensive review).

Our experimental design included both free recall and forced-choice recognition memory tests to examine differences between the two memory processes. It is important to consider that forced-choice recognition carries a higher risk of random guessing, which tends to increase as performance declines^64^. Consistent with expectations, forced-choice performance was indeed better than free recall performance. As hypothesized, the interaction between response modality and cognitive load revealed a higher dual-task cost for free recall compared to forced choice.

Unexpectedly, for the memory task, we did not find a significant interaction between response modality and age, nor among response modality, age, and cognitive load. This suggests a common impact on both recollection-based and familiarity-based processes. Additionally, we observed that younger participants performed near ceiling levels in the forced-choice response modality. The visual Memory task effectively triggered the need to prioritize one task over another as indexed by the negative correlation (trade-off) between costs in visual Memory and in verbal Fluency. The correlations between performance in TMT/memory with interference and dual-tasking cost in the visual Memory task link the ability of splitting attention to concurrently process visual and verbal material across very different task, in the absence of a significant correlation with MoCA. In this respect, while the MoCA provides a general estimate of the participant’s cognitive efficiency, the other tasks measure specific cognitive abilities (working memory and executive functions) which are likely closer to those cognitive constructs subtending multitasking^8^. Taking this into account, the cost in our computer-based memory task index could be a potential sensitive indicator of individual variation in those multitasking skills that are fundamental to cognitive efficiency in healthy individuals.

According to the Life-Span perspective^65^, the ageing process accompanies the individual throughout the entire life span and is defined as an active and constant search for balance between gains and losses where the loss of neural efficiency could be compensated by optimisation of cognitive mechanisms resulting in normal mnestic performance. As mentioned previously, executive functions/WM are the central constructs of many neuropsychological tests that aim to differentiate between normal and pathological ageing^66,67^. Our results show that while maintaining the typical characteristics of a memory test, the visual Memory dual-task can simultaneously capture the central executive efficiency which plays a key role in our daily multitasking activities.

### Variability in the memory task

A final aspect to be taken into account is performance variability. As previously reported, Levene’s test revealed a greater variability in cost for free recall than for forced choice. In a memory task with a binomial dependent variable, mean and standard deviation are expected to be strongly associated (covariate). As shown in Table 2, the forced-choice single task had the highest mean performance, with an average probability of 0.92 across all ages for remembering an item, and exhibited the lowest variability, potentially indicating a ceiling effect. Surprisingly, despite very similar (and far from ceiling) average accuracy, the dual-task forced choice showed higher variability than the single-task free recall. This suggests that individuals had varying levels of susceptibility to cognitive load. Individual susceptibility to cognitive load may be influenced by the strategies employed (preference for one task over the other), as well as individual differences in the availability of resources, as the coping capacity for dual tasks is challenged. This second hypothesis is also supported by the correlation between cost in the memory task and the Delta TMT score which might suggest that these indexes are sensitive markers of performance deterioration. Further support to this possibility comes from a recent study by our research group^28,30^, which revealed that participants who incurred the greatest costs during visuo-attentional dual-tasking exhibited significantly lower scores on a cognitive screening test compared to their peers. In that study, we coupled a visual primary task similar to the one used here but without the free recall condition and with a concurrent task based on auditory sustained attention. Drawing on our previous experience, we calibrated that task in such a way that it was sufficiently responsive to age variations, while not being too difficult for older individuals or too simple for younger individuals. The web-based battery of tests was self-administered and responses were collected through an automatic procedure which would make this approach ideal for longitudinal monitoring.

However, for future task developments, it would also be important to consider the relative contribution that recalling and familiarity have in the retrieval process and how this impacts different memory tasks and individuals^43^. Although one might expect a higher cost for free recall, the more challenging task that relies primarily on recollection, it is also possible that some participants perform worst on forced choice, an easier task that relies mostly on familiarity. The latter pattern could be due to increased susceptibility to the presence of salient distractors, a typical feature of normal ageing^68,69^, which is greatly amplified in the case of dementia^70–72^. In the current study, the sample size was not sufficiently large to reliably study the presence of possible clusters of participants. Future studies with larger samples should precisely target individual variability across forced choice and free recall in a dual memory task as a possible source of information for the early detection of the first signs of cognitive decline.

### Clinical relevance

Neurodegenerative diseases are highly prevalent among older adults and in many cases can remain undetected for long as persons in prodromal stages of degeneration show few or no evident symptoms which can sometimes nonetheless still be detectable using sufficiently sensitive tests^73^. At present, such patients are often referred to clinical examination when their cognitive decline is very severe, and their clinical picture has become not only incontrovertible but also unmodifiable. At this advanced stage, treatments such as cholinesterase inhibitors offer only modest benefits in alleviating symptoms and slowing disease progression^74^. For this reason, pharmacological and neuropsychological research has made early prevention and treatment more attainable^75^. The ideal goal would be to promote an early intervention – resembling a prevention – significantly before dramatic cognitive disorders are evident^76–78^. Recently, the FDA approved the use of the monoclonal antibody Aducanumab for the treatment of Alzheimer’s^79^. Aducanumab has been shown to be effective in clearing extracellular amyloid beta plaques observed in AD and in reducing cognitive decline^80^ as measured by standard paper and pencil tests. Anticipating the detection of a potential deficit would allow activating psychological and pharmacological support before the appearance of obvious clinical signs, ideally increasing the chances of postponing the severe stages of the disease. This study was exploratory in nature, primarily focused on assessing general cognitive performanc across different ages. It seems then important to enphasize that we are still far from a clinical implementation. However, a computerized CCDT such as the one presented in this study could be a preliminary step for developing a tool for better monitoring and preventing cognitive efficiency in older adults. One particularly promising option seems to longitudinally administer such tasks from remote^28,30^ while using countermeasures to avoid learning effects. It is worth emphasizing that we are still far from a comprehensive understanding of the optimal combination of tasks indexes to be used as future indicators of cognitive decline to arrive. Nonetheless, by contrasting performance data from a variety of tasks, we can identify the indexes that provide the most useful information. Additionally, it is crucial to identify tasks like the one described here which linearly deteriorate due to age and cognitive load, as they can serve as a reliable benchmark against which to compare other tests.

## Data Availability

The experimental design and analyses of the first experiment were pre-registered on Open Science Framework (OSF) before data collection. The images used in the memory task, and datasets generated and/or analysed during the current study are available in the OSF repository.
